# Is Empiricism Innate? Preference for Nurture Over Nature in People’s Beliefs About the Origins of Human Knowledge

**DOI:** 10.1162/opmi_a_00028

**Published:** 2019-09-01

**Authors:** Jinjing (Jenny) Wang, Lisa Feigenson

**Affiliations:** Department of Psychological and Brain Sciences, Johns Hopkins University; Department of Psychological and Brain Sciences, Johns Hopkins University

**Keywords:** nature-nurture, intuitive theories, core knowledge, nativism, empiricism

## Abstract

The origins of human knowledge are an enduring puzzle: what parts of what we know require learning, and what depends on intrinsic structure? Although the nature-nurture debate has been a central question for millennia and has inspired much contemporary research in psychology and neuroscience, it remains unknown whether people share intuitive, prescientific theories about the answer. Here we report that people (*N* = 1,188) explain fundamental perceptual and cognitive abilities by appeal to learning and instruction, rather than genes or innateness, even for abilities documented in the first days of life. U.S. adults, adults from a culture with a belief in reincarnation, children, and professional scientists—including psychologists and neuroscientists, all believed these basic abilities emerge significantly later than they actually do, and ascribed them to nurture over nature. These findings implicate a widespread intuitive empiricist theory about the human mind, present from early in life.

## INTRODUCTION

Some questions about human nature persistently captivate our thinking. Among these is the puzzle of where knowledge comes from: what must be learned from our actions and observations, and what emerges from our biological inheritance? This mystery of how nature and nurture give rise to the mind has animated debate for millennia (Cooper & Hutchinson, 1997), inspiring research in linguistics, neuroscience, and psychology. Although there is consensus that some mental abilities are built from experience and instruction, like reading and calculating square roots, there is disagreement over what innate capacities exist in support of this learning. Arguments that some capacities depend on innate structure point to findings of competence in very young infants or individuals lacking relevant learning opportunities (Izard, Sann, Spelke, & Streri, 2009; Senghas, Kita, & Özyürek, 2004), and to the observation that even with such opportunities, the available data underspecify what humans seem to learn (Chomsky, 1980). These arguments face counterclaims that this knowledge instead is acquired via powerful learning mechanisms, sometimes soon after birth (Skinner, 2014; Verguts & Fias, 2004). Although the nature-nurture debate continues, the empiricist position that knowledge requires learning has dominated much of the last century (see Margolis, Samuels, & Stich, 2012), with some arguing that empiricism should be the default position absent undeniable evidence otherwise (Prinz, 2012).

In this ongoing debate between nativism and empiricism, scientists use the mind to study the mind. But what if the primary instrument in this endeavor, the human mind, intuitively favors one explanation of knowledge over the other? Children and adults hold a variety of intuitive theories that are sometimes at odds with (and may later be replaced by) theories acquired through formal instruction (Carey, 2009). These intuitive theories are potent enough that they sometimes affect scientific theorizing by professionals. An example comes from the science of object physics. For over a thousand years, from Philoponus in the 6th century to Galileo in the 16th, philosopher-scientists mistakenly believed that moving objects acquire an energy that propels them until the energy runs out (McCloskey, 1983). Their “impetus theory,” incompatible with Newtonian mechanics and contemporary physics, is not just an archaic view. Modern adults also make wrong predictions about object motion; strikingly, their predictions echo those of the premedieval and medieval scientists, even though they were never taught the impetus theory and had even taken college physics (McCloskey, 1983). This shows that beliefs about the world, even formal scientific beliefs held by specialists, can reflect folk intuitions that are broadly shared and resistant to revision. With this in mind, consider again the case of where basic human knowledge originates. For this ancient question that continues to drive current research, generating answers from the dichotomous (“nature or nurture”) to the more nuanced (“nature and nurture”), do people think of nature and nurture as natural alternatives? If so, do they intuitively prefer one as the better explanation?

To characterize folk beliefs about nature and nurture, we probed intuitions about aspects of the mind that have been studied experimentally, thereby providing a “ground truth” against which to compare. The basic, nonverbal, “core” knowledge that guides everyday actions—like believing that hidden objects still exist, or that 10 apples are more than five, offers such a case, because research of the past 40 years has carefully examined its developmental origins. Laboratory experiments have illuminated ways in which infants represent and reason about objects, quantities, and other people by measuring their looking times and neural responses to these stimuli (Spelke & Kinzler, 2007). Comparing what science has discovered about the origins of this foundational core knowledge to people’s intuitive theories can reveal both hidden systematicities and limitations in thinking about thinking.

## EXPERIMENT 1

### Participants

One hundred and one adults (*M* = 34.95 years old; 59 female) were recruited through Amazon Mechanical Turk.

### Design, Stimuli, and Coding

Participants read a description of a character Alex, with four of her behaviors chosen to illustrate an innate ability, an ability emerging through biological maturation, an ability learned through observation and practice, and an explicitly taught ability: (1) Alex was born able to drink milk, (2) Alex grew older and became strong enough to lift heavy things, (3) Alex learned on her own how to play on a computer, (4) Alex learned from someone else how to tie her shoes. Then participants were presented seven core perceptual and cognitive abilities, each the subject of much previous developmental research: color perception (distinguishing two colors), depth perception (distinguishing a nearby from a distant object), face recognition (distinguishing facelike from non-facelike things), physical reasoning (thinking an unsupported object will fall), object permanence (thinking a hidden object still exists), approximate numerical discrimination (thinking an array of 10 items has more than an array of five), and social evaluation (preferring helping others to not helping) (Baillargeon, 1987, 1995; Bornstein, Kessen, & Weiskopf, 1976; Farroni et al., 2005; Hamlin, Wynn, & Bloom, 2007; Izard et al., 2009; Slater, Mattock, & Brown, 1990) (Table S1, Wang & Feigenson, 2019). Participants indicated when they thought each ability first was present by clicking an image depicting Alex as a newborn, older infant, toddler, preschool-age child, school-age child, adolescent, or adult. Participants rarely chose these last two images; therefore, we excluded them from the figures’ *x*-axes but included any responses to them in all analyses. Participants also were asked “how come” Alex had each ability, and typed a free response. To confirm that participants understood the task, we included three anchor items involving abilities predicted to generate consensus as being present early in life and requiring little or no learning (seeing and hearing), or as appearing later and requiring learning and instruction (reading).

**Figure 1.**
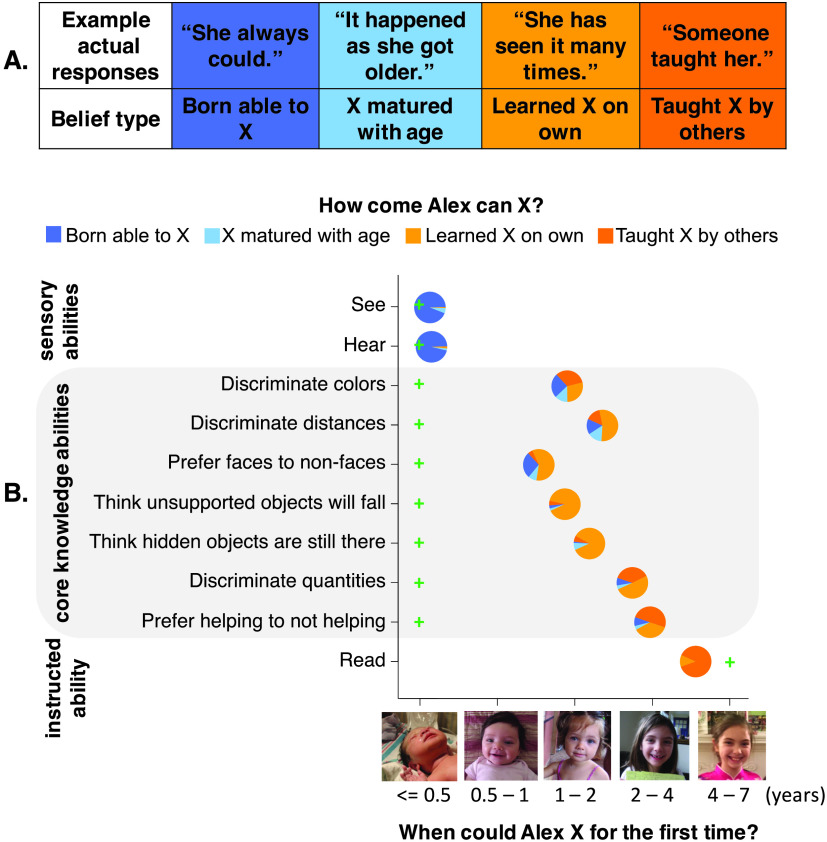
**Methods and results for Experiment 1.** (**A**) Example responses to “How come Alex can [tell colors apart]?” and categories of coded responses. (**B**) Mean responses in Experiment 1. Circles’ left-right positions represent participants’ age estimates. Circles’ colors represent the distribution of free responses. Green crosses indicate the earliest age category at which abilities have been documented in published research.

We quantified participants’ timeline choices by first estimating Alex’s age in each timeline image using the midpoint between the labeled lower and upper age bounds (Figure 1B). We fit these ages using their ordinal positions, resulting in the function *y* = 0.13*e*^0.78*x*^, *R*^2^ > .99. Using this equation, we translated participants’ timeline responses into an average age onset estimate for each ability, allowing us to compare participants’ estimates to the average onset age suggested by published findings (Table S2, Wang & Feigenson, 2019).

Participants’ free responses to the question “how come Alex can X” were coded into four categories (Figure 1A). Explanations that an ability “was innate” or was due to biological structure (e.g., “she can see because she has eyes,” “it’s in her genes”), or that a person “was just born able to X” were coded as endorsing innateness. Explanations that an ability “emerged on its own” or “happens as a person gets older” were coded as endorsing maturation. Explanations that a person “became able to X through their own observations,” or “learned X on their own,” were coded as endorsing learning without explicit instruction. Explanations that an ability “was taught by others” or “was learned in school” were coded as endorsing explicit teaching. Of 1,010 responses, 15% did not clearly fall into any category (e.g., “kids have this ability at this age,” “she is smart”), and were excluded from analysis.

### Results

Participants estimated that core knowledge abilities emerged, on average, between 0.94 and 2.88 years of age (Figure 1B) (95% CIs [0.66, 1.22] [2.60, 3.15]), more than eight times the average age of onset suggested by published findings. Strikingly, participants overwhelmingly explained the core abilities using learning-based explanations (explanations endorsing learning without instruction plus explanations endorsing explicit teaching), at levels exceeding chance (*M* = 77%; 95% CI [73%, 81%]; *t*(99) = 11.81, *p* < .001) (Figure 1B). For no core ability did learning-based responses drop below 50%, even for the more perceptual abilities of distinguishing colors (62%) and distances (69%; binomial exact tests *p*s < .05). For the remaining core abilities, learning-based responses were even more prevalent, averaging 82%; 95% CI [77%, 87%] (see the Supplemental Materials, Wang & Feigenson, 2019, for analyses of the influence of gender, age, educational background, parental status, and relevant coursework on participants’ responses). Hence, adults believed that basic perceptual and cognitive abilities, suggested by research to be available in early infancy, are acquired much later through learning and instruction.

This was not because participants simply pointed to images of older children, or did not understand the idea of innateness. For seeing and hearing, adults correctly believed that infants do both (*M* = 0.32 years; 95% CI [0.05, 0.58]) (Brown & Yamamoto, 1986; Northern & Downs, 2002), and almost never offered learning-based explanations (*M* = 3%; 95% CI [0%, 5%]). For reading, adults believed that this ability appears around early school age (*M* = 4.56 years; 95% CI [4.29, 4.83]) (Hasbrouck & Tindal, 2011), and 100% gave learning-based explanations.

Two replication studies tested alternative explanations of our findings, confirming that the observed pattern was not due to participants’ perception of the timeline, nor to the criteria used to code free responses. Participants in Experiment 1b (*N* = 100) were probed on the same test items but, for each ability, typed the age at which they thought each ability first was present (without a timeline), and indicated the ability’s origins by adjusting a sliding bar in response to the prompt “Where does Alex’s ability to [e.g., tell colors apart] come from?” The bar’s endpoints were 0 (“entirely from her genes”) and 100 (“entirely from her experience”). Participants in Experiment 1c (*N* = 202) were tested with the timeline and free response, using ability descriptions with increased or decreased emphasis on metacognitive or verbal capacities. For example, instead of “tell colors apart,” we asked “When was the first time/how come Alex’s brain [responded differently to different colors]?”). In both replications, participants again overestimated the core abilities’ onset, and attributed them to learning (Figures S1 and S2, Wang & Feigenson, 2019).

Is the intuitive empiricism revealed in Experiment 1 culturally specific? To find out, we tested adults living in India, where approximately 80% of people identify as Hindu. A major tenet of Hinduism involves reincarnation, a belief that could increase nativist beliefs because aspects of the self are thought to precede individual experience.

## EXPERIMENT 2

### Participants

Ninety-nine adults living in India, who self-identified as Hindu (*M* = 29.49 years; 22 female), were recruited through Amazon Mechanical Turk.

#### Design, Stimuli, and Coding

All aspects were as in Experiment 1. In addition, participants reported their religiosity on a 5-point scale (1 = very religious, 5 = not at all religious). Of 990 free responses, 36% did not fall into any category and were excluded.

### Results

**Figure 2.**
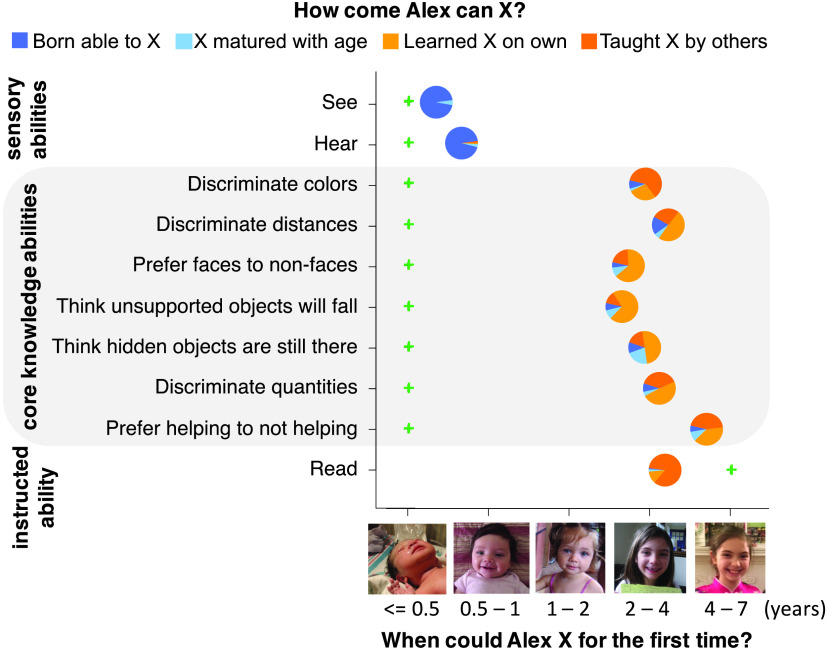
Mean responses in Experiment 2.

Like U.S. participants, Indian participants believed core abilities emerge later than they actually do, choosing onsets between 2.25 and 5.11 years (95% CIs [1.98, 2.53] [4.84, 5.38]), more than 16 times the age of actual onset, and gave mostly learning-based explanations of the abilities’ origins (*M* = 80%; 95% CI [74%, 86%]) (Figure 2). Degree of religiosity predicted neither onset estimates (*β* = −0.14, *p* = .20) nor learning-based explanations (*β* = 0.032, *p* = .77).

Are the intuitively empiricist beliefs of U.S. and Indian adults specific to human abilities, or is it how people think about minds generally, including those of other creatures? Research on nonhuman species has revealed core abilities similar to those in humans, allowing a variety of creatures to represent objects, quantities, and other animals (Spelke & Kinzler, 2007). Experiment 3 compared beliefs about human versus animal knowledge.

## EXPERIMENT 3

### Participants

Two hundred and one adults (*M* = 33.23 years; 136 female) were recruited through Amazon Mechanical Turk.

### Design, Stimuli, and Coding

We asked participants about core abilities in humans (*N* = 100) or other animals (*N* = 101). As before, we included anchor abilities chosen to elicit agreement that learning was either not required (seeing in humans/horses) or was required (washing hands [humans]; using a litter box [cats]). The critical test items were core perceptual and cognitive abilities demonstrated in both humans and animals: humans/chickens discriminating faces of conspecifics from nonfaces (Farroni et al., 2005; Rosa-Salva, Regolin, & Vallortigara, 2010), humans/spiders discriminating nearby from distant objects (Nagata et al., 2012; Slater et al., 1990), humans/salamanders discriminating colors (Bornstein et al., 1976; Przyrembel, Keller, & Neumeyer, 1995), humans/ants discriminating angles of rotation (Landau & Spelke, 1988; Müller & Wehner, 1988), humans/crows following others’ gaze (Johnson, Slaughter, & Carey, 1998; Schloegl, Kotrschal, & Bugnyar, 2007), humans/bees discriminating two objects from three (Gross et al., 2009; Starkey & Cooper, 1980), humans/fish discriminating approximate numerosities (Agrillo, Dadda, Serena, & Bisazza, 2008; Izard et al., 2009), and humans/chickens thinking a hidden object still exists (Baillargeon, 1987; Vallortigara, Regolin, Rigoni, & Zanforlin, 1998). Participants reported whether they believed each ability to be present at birth, and described its origins using free response. Coding and analysis were as in Experiment 1. Of 2,010 free responses, 18% did not fall into any category and were excluded.

### Results

**Figure 3.**
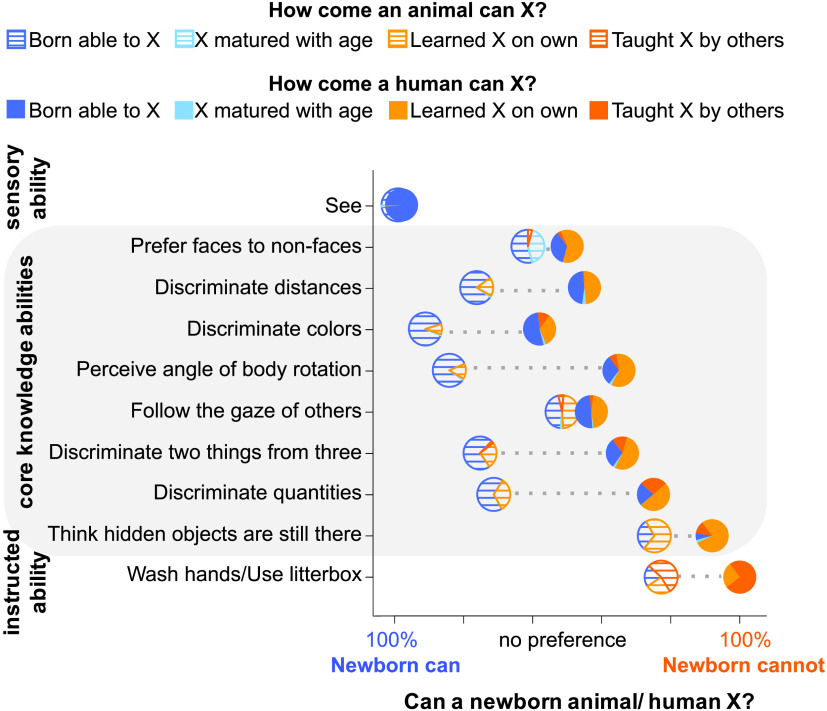
Mean responses in Experiment 3.

People’s intuitions about the anchor abilities were similar for humans and animals: Adults reported seeing as present at birth in both species (human *M* = 98%; horse *M* = 99%), and almost never offered learning-based explanations (human *M* = 1%; horse *M* = 1%). They believed that washing hands/using a litter box is not present at birth in either humans or cats (human *M* = 0%; cat *M* = 23%), and offered mostly learning-based explanations (human *M* = 100%; cat *M* = 79%) (binomial exact test *p*s < .001). Critically, we found that people’s intuitions diverged for the core ability items: they were significantly less likely to endorse core abilities as present in newborn humans (*M* = 37%; 95% CI [32%, 43%]) than newborn animals (*M* = 67%; 95% CI [63%, 71%]; *t*(199) = 8.43, *p* < .001), and offered more learning-based explanations for core abilities in humans (*M* = 62%; 95% CI [56%, 68%]) than animals (*M* = 31%; 95% CI[26%, 36%]; *t*(198) = 7.69, *p* < .001); this difference was significant for 7/8 core items (*χ*^2^s > 4.76, *p*s < .029) (Figure 3). Whereas people readily explained animals’ abilities by appeal to genes, evolution, and innateness, for the very same abilities in humans they typically invoked observation and learning.

## EXPERIMENT 4

Experiments 1–3 suggest a widespread belief that basic human abilities require learning. That people did not show this commitment about animals further confirms that this pattern was not a methodological artifact. But where does this intuitive theory come from? It might form slowly, perhaps with years of education highlighting learning and instruction. Alternatively, it might be in place early in life. To find out, in Experiment 4 we tested children.

### Participants

Eighty-five children (*M* = 6.68 years, *SD* = 1.01; 62 female) were tested at a science museum.

### Design, Stimuli, and Coding

Children heard a description of Alex, followed by four examples of Alex’s abilities highlighting innateness, maturation, observational learning, and learning through instruction (see Experiment 1). Next, children were asked about the abilities from Experiment 1 (two sensory abilities, seven core knowledge abilities, reading). Items were presented verbally, with props to illustrate (e.g., cookies with different numbers of chocolate chips to illustrate distinguishing more from less). Children used the timeline to indicate the age at which they thought abilities first were present, and were asked “how come” Alex had each ability. If children refused to answer or gave an uninterpretable response, the experimenter asked a series of follow-up questions. For example, if children did not answer “How come Alex can see?” the experimenter asked, “Could Alex see the first time she opened her eyes, or did Alex need to open her eyes many times to be able to see?” If children answered “many times,” the experimenter asked, “Did she learn it on her own, or did someone have to teach her?” If children responded that Alex could do X the first time, this was coded as endorsing innateness. If children said Alex needed many times before she could X but that no one taught her, this was coded as endorsing learning through observation. If children said someone taught her, this was coded as endorsing explicit instruction.

Children’s responses were transcribed and coded. Of 514 responses, 57% did not initially fall into any defined category, in which case children were asked the follow-up questions.

### Results

**Figure 4.**
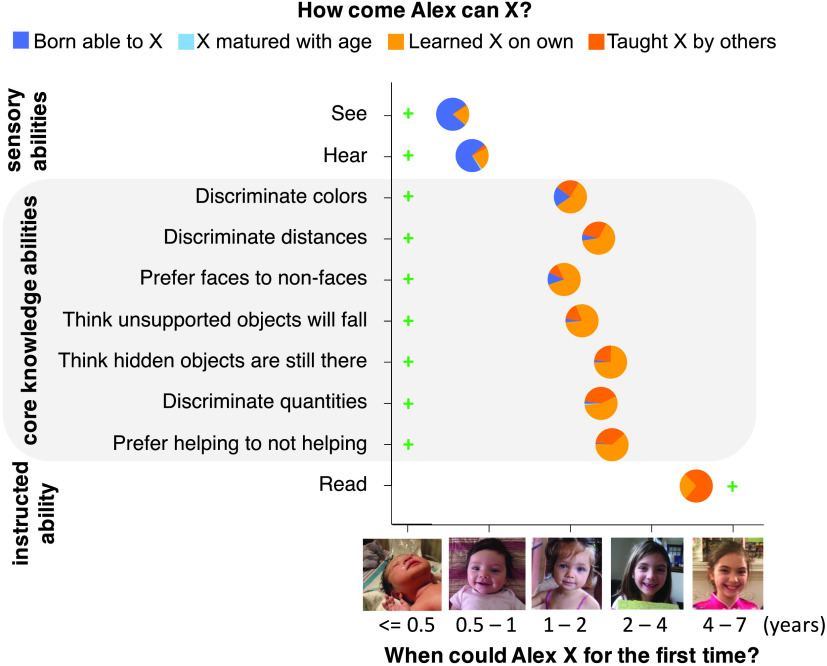
Mean responses in Experiment 4.

Children overestimated the onset of core knowledge abilities, citing them as emerging between 1.27 and 2.01 years (95% CIs [0.99, 1.55] [1.74, 2.29]), more than eight times the onset suggested by published findings. Children overestimated more for the core abilities than sensory and reading anchor abilities, *t*(84) = 10.08, *p* < .001; *t*(83) = 5.29, *p* < .001. Like adults, children overwhelmingly gave learning-based explanations of the core abilities’ origins (*M* = 92%; 95% CI [89%, 95%], *t*(84) = 25.52, *p* < .001), rarely invoking innateness (Figure 4). Indeed, children gave more empiricist explanations of the core abilities than adults in Experiment 1, *t*(183) = 5.29, *p* < .001. This was not due to vocabulary limitations; like adults, children reported that sensory abilities (seeing and hearing) do not require learning (*M* = 24%; 95% CI [17%, 32%]).

## EXPERIMENT 5

Experiments 1–4 show that adults and children think of foundational aspects of human knowledge as taking years to emerge and deriving from experience. Is this belief shared by scientists, including those who study the biological and psychological foundations of the mind? In the case of the impetus theory, medieval philosopher/scientists held beliefs similar to those of modern nonspecialist adults. To find out whether contemporary scientists share the commitment that core human abilities are mostly learned, in Experiment 5 we tested professional academics.

### Participants

Participants were 400 faculty, postdocs, and graduate students from 12 geographically diverse U.S. universities (*M* = 41.80 years; 272 female): 100 in natural sciences (e.g., chemistry, astronomy), 100 in humanities (e.g., literature, gender studies), and 200 in mind sciences (e.g., psychology, neuroscience, linguistics). Thirty-six percent reported having completed a PhD.

### Design, Stimuli, and Coding

All aspects were as in Experiment 1. Of 4,000 free responses, 7% did not fall into any defined category and were excluded.

### Results

**Figure 5.**
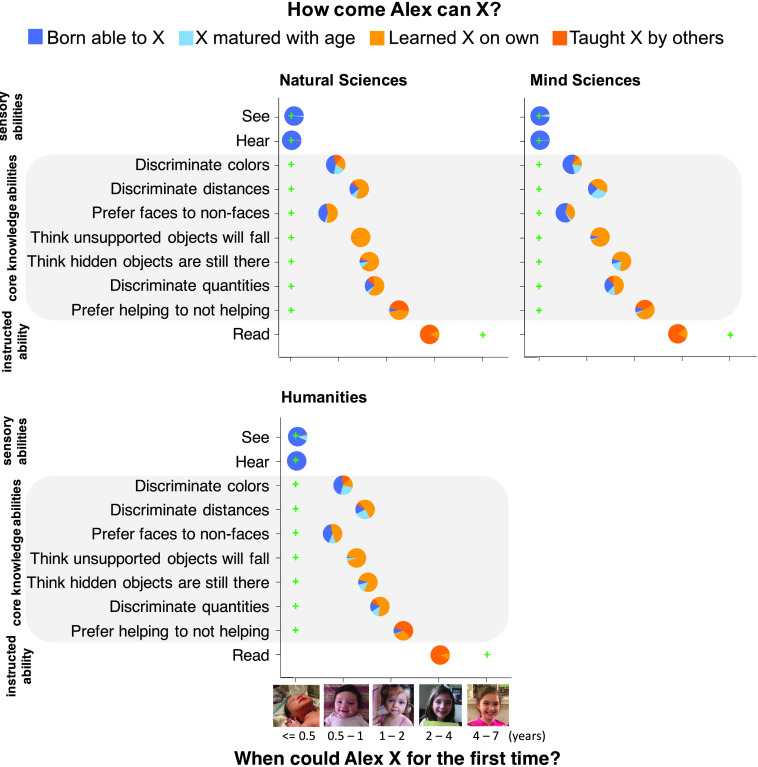
Mean responses in Experiment 5.

Academics overestimated the core abilities’ age of onset, citing them as first available between 0.48 and 1.63 years (95% CIs [0.21, 0.74] [1.37, 1.89]), more than four times the age suggested by published findings. Like children and nonspecialist adults, they gave predominantly learning-based explanations of the abilities’ origins (*M* = 64%; 95% CI [62%, 67%]) (Figure 5). However, they offered fewer learning-based explanations than the nonspecialist participants in Experiment 1, *t*(497) = 4.76, *p* < .001, and mind scientists offered fewer learning-based explanations than participants in the natural sciences and humanities, *t*(399) = 4.32, *p* < .001. These results suggest that education may attenuate people’s preference for empiricist explanations. Nonetheless, even mind scientists alone, whose area of expertise included perceptual and cognitive abilities like those in our survey, preferred to give explanations invoking nurture over nature (learning-based explanations *M* = 59%, 95% CI [55%, 63%]).

## GENERAL DISCUSSION

For thousands of years humans have pondered the origins of our mental lives, debating the extent to which aspects of our knowledge rely on nature versus nurture. More recently this dialogue has moved into the laboratory, with scientists from diverse disciplines seeking to uncover what arises in the absence of specific experience versus what must be learned. Our experiments reveal that naïve adults in the United States and India, children, and highly educated adults, including psychologists and neuroscientists, all share the intuition that core perceptual and cognitive abilities take years to emerge (including abilities for which research suggests otherwise), and are mostly learned. Whether this widespread belief is right or wrong is a matter for continued scientific discovery, although in the cases tested here there is compelling evidence that human minds have at least some early structure. Therefore, although a dichotomous understanding of the nature/nurture debate is surely too simple to be correct (e.g., because nature always must anticipate a particular environment in which development will occur), some of the beliefs of human thinkers appear to run contrary to our current science.

Where does this intuitive theory of human abilities come from? One possibility, to share a classic quip by psychologist and linguist Lila Gleitman, is that “empiricism is innate.” Although Gleitman may have made the suggestion tongue in cheek, a bias to focus on learning as the source of knowing could conceivably be a product of evolution, selected for because it increases pedagogy and encourages information transmission between individuals (Csibra & Gergely, 2011). Our data are at least consistent with this hypothesis, in that we observed empiricist intuitions in the youngest children tested. Alternatively, intuitive empiricism might be learned—perhaps by noticing the enormous effort and resources humans spend on teaching (Legare, 2017), by seeing that infants are behaviorally limited, or observing that many human abilities (like reading) do require experience and practice.^1^ Such observations could foster the conclusion that humans lack knowledge until later in life, after experience and instruction have accrued. If evidence for pedagogy or developmental differences in competence are lacking in people’s experience of nonhuman animals, this could help explain the divergence in people’s intuitions about human versus animal abilities. Finally, it is possible that people’s preference for empiricist explanations is promoted by the feeling that focusing on learning is more optimistic than the alternative. A belief that knowledge is acquired could lead people to conclude that with relevant experience anything can be learned; this in turn could generate a sense of equality among individuals. Future work should test these possibilities.

In addition, it will be important for future research to test the scope of intuitive empiricism. For aspects of human nature other than the basic perceptual and cognitive abilities tested here, adults and children often implicate an underlying nature rather than experience—a phenomenon termed *essentialism* (Gelman, 2004). For example, people typically believe that members of categories defined by gender or ethnicity differ in an inherent, unlearned way (Prentice & Miller, 2007). And when adults are asked about the origins of many human temperament traits (like positive affect), they less often implicate learning and more often appeal to innate disposition (Haslam, Bastian, & Bissett, 2004). It is against this backdrop that people’s commitments about perception and cognition are so remarkable: we seem to believe that much of what we know derives from experience, but that much of who we are is rooted in biology.

An inspiration for this research was the recognition that science is held as a paradigm of inquiry in which evidence—not preference or prejudice—adjudicates between theories. Yet because science is an endeavor of human minds, and because minds tend to reason about the world in systematic ways (e.g., Tversky & Kahneman, 1974; Vosniadou & Brewer, 1992), our intuitive theories unavoidably play a role in forming and evaluating scientific theories (Bloom & Weisberg, 2007). As such, our findings highlight a key role for psychological research throughout science: psychology can and should uncover intuitive theories that shape everyday thought, and that also may affect inquiry across scientific disciplines, from physics to biology to psychology itself.

## ACKNOWLEDGMENTS

We thank the families who participated and the Maryland Science Center. We thank C. Firestone, S. Gross, and J. Halberda for comments, V. Amandan, K. Carosella, A. McManus, D. Osaji, M. Shah, and A. Silver for assistance with data collection and coding, and L. Gleitman for initial inspiration and discussion.

## AUTHOR CONTRIBUTIONS

JW: Conceptualization: Equal; Data curation: Lead; Formal analysis: Equal; Methodology: Equal; Visualization: Equal; Writing - Original Draft: Equal; Writing - Review & Editing: Equal. LF: Conceptualization: Equal; Formal analysis: Equal; Methodology: Equal; Supervision: Lead; Visualization: Equal; Writing - Original Draft: Equal; Writing - Review & Editing: Equal.

## Note

^1^ Noticing that some abilities improve over time might lead participants to report the age at which the ability is mature, rather than its age of first onset. To steer participants away from responding based the on age of abilities’ maturity, we always asked when each ability was present “for the first time.” Participants’ systematic underestimation of the age of reading onset suggests that they were not reporting their beliefs about the age of asymptotic performance, since reading is an ability that undergoes many years of formal instruction and improvement.
