# Supplementary material for: Is Empiricism Innate? Preference for Nurture Over Nature in People’s Beliefs About the Origins of Human Knowledge
Source: Open Mind (Camb). 2019 Sep 1;3:89–100. doi: 10.1162/opmi_a_00028 (PMC8412204; doi:10.1162/opmi_a_00028)
Supplement: Supplementary file 1 [file opmi-03-89-s001.pdf]

**Is Empiricism Innate? Preference for Nurture over Nature in People's Beliefs about the Origins of Human Knowledge**

**Supplemental Material**
https://doi.org/10.1162/opmi_a_00028

**Authors:** Jinjing (Jenny) Wang and Lisa Feigenson

**Author Affiliations:** Department of Psychological and Brain Sciences, Johns Hopkins University, 3400 N. Charles Street, Baltimore, MD 21218 USA

**Corresponding Authors:** Jinjing (Jenny) Wang, jinjing.jenny.wang@gmail.com

## Experiment 1

Additional Coding Information

A second observer coded 35% of all data. Inter-coder reliability was 99%.

Additional Results

In addition to the results reported in the main text, we examined the relationship between participants' responses to the core knowledge items and their gender, age, level of education, parental status, and whether they had taken any developmental psychology courses.

A univariate linear regression with participants' Age Onset Estimates as outcome variable and Gender, Age, Education, Parental Status, and Relevant Coursework as predictors

revealed that Gender was a significant predictor (β = -0.24, $p$ = .02), with male participants offering earlier age onset estimates (*M*: 1.94 years, 95% CI [1.65, 2.23]) than female participants (*M*: 2.41 years, 95% CI [2.16, 2.66]). Age (β = 0.19, $p$ =.07), Education (β = -0.03, $p$ =.78), Parental Status (β = -0.02, $p$ =.85), and Relevant Coursework (β = -0.07, $p$ =.48) did not predict participants' Age Onset Estimates. A univariate linear regression with percentage of Learning-based Explanations as outcome variable and Gender, Age, Education, Parental Status, and Relevant Coursework as predictors revealed Gender (β = -0.30, $p$ = .004) as the only significant predictor; male participants offered fewer learning-based explanations (*M*: 69%, 95% CI [61%, 76%]) than female participants (*M*: 82%, 95% CI [77%, 88%]). Age (β = -0.02, $p$ =.84), Education (β = 0.07, $p$ =.50), Parental Status (β = -0.07, $p$ =.50), and Relevant Coursework (β = -0.08, $p$ =.45) did not predict participants' Learning-based Explanations.

**Experiment 1b**

To ask whether participants' judgments in Experiment 1 were caused by the way they perceived the photographs in the response timeline, or by our criteria for coding their free responses, we replicated Experiment 1 using new measures for both age onset estimates and beliefs about the origins of each ability. Participants typed age estimates for ability onsets rather than choosing among photographs, and used a sliding scale to indicate the extent to which they thought each ability was learned, rather than typing a free response.

Method

Participants

100 adults (*M*: 34.7 years; 57 female) were recruited online through Amazon Mechanical Turk.

Design and stimuli

Materials were largely as in Experiment 1, with new dependent measures. To assess participants' beliefs about the age of onset of the various abilities, participants were asked to type the earliest age Alex first had each ability. Response boxes were provided for age in years and months and participants could use either or both. No timeline photographs were shown. Participants indicated their beliefs about the abilities' origins by adjusting the position of a sliding bar in response to the prompt "Where does Alex's ability to [e.g., tell colors apart] come from?" The endpoints of the bar were 0 ("entirely from her genes") and 100 ("entirely from her experience"); participants could indicate any position between these.

Results

The results replicated Experiment 1. Participants overestimated the core abilities' age of onset (*Ms*: between 1.77 and 3.75 years; 95% CI [1.46, 2.09] [3.23, 4.27]), and invoked experience over genes as their cause (*M*: 66%; 95% CI [62%, 70%]) (Fig. S1).

In addition, we examined the relationship between participants' responses on the core knowledge items and their gender, age, level of education, and parental status. A univariate linear regression with Age Onset Estimates as outcome variable and Gender, Age, Education, and Parental Status as predictors revealed no significant predictors: Gender ($\beta$ = 0.20, $p$ = .06), Age ($\beta$ = -0.08, $p$ =.43), Education ($\beta$ = -0.05, $p$ =.61), Parental Status ($\beta$ = 0.06, $p$ =.55). A univariate linear regression with percent of learning indicated as outcome variable and Gender,

Age, Education, and Parental Status as predictors also revealed no significant predictors: Gender ($\beta$ = 0.009, $p$ = .93), Age ($\beta$ = -0.04, $p$ =.69), Education ($\beta$ < 0.001, $p$ > .99), Parental Status ($\beta$ = 0.14, $p$ =.18).

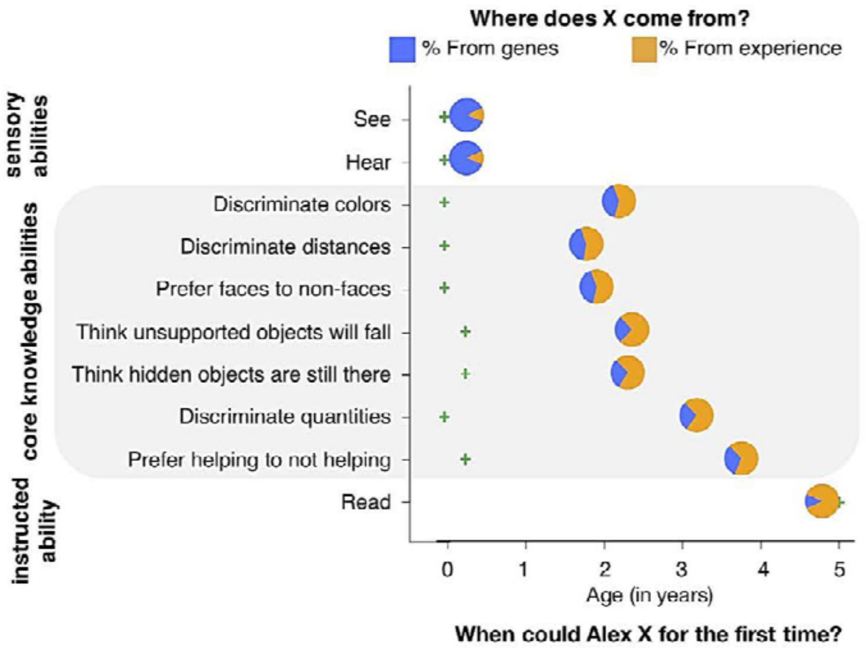


**Fig. S1.** Mean responses in Experiment 1b. Green crosses indicate the earliest age (in years) at which abilities have been documented in published research.

**Experiment 1c**

Participants in Experiments 1 and 1b overestimated the age of onset of core knowledge abilities, and overwhelmingly appealed to learning and experience to explain the abilities' origins. Was this due to the way we worded the items, including our use of the verbs "tell" and

"think," which could have connoted metacognitive awareness or verbal proficiency (e.g., "When could Alex [tell colors apart] for the first time?")? Experiment 1c replicated Experiment 1 using descriptions that increased or decreased the emphasis on metacognitive or verbal capacities.

## Method

### Participants

202 adults (*M*: 35.59 years; 108 female) were recruited through Amazon Mechanical Turk.

### Design and stimuli

Design and stimuli were as in Experiment 1, with modified wordings (Table 3). Adults either saw abilities described in terms of behaviors (e.g., "When could Alex [see the difference between colors] for the first time?") (N = 50), brain activity (e.g., "When was the first time Alex's brain [responded differently to different colors]?") (N = 51), epistemic states (e.g., "When could Alex [know how to tell colors apart] for the first time?") (N = 50), or using the wording from Experiment 1 ("When could Alex [tell colors apart] for the first time?") (N = 51).

### Coding and Analyses

Coding and analyses were as in Experiment 1. Inter-coder reliability was 97%. Out of 2020 free responses, 15% did not clearly fall into any of the defined categories and therefore were excluded from analysis.

## Results

Participants chose similar age onsets across the four wording conditions (Fig. S2); a 4 (Wording: Behavior, Brain, Epistemic, Tell) x 3 (Knowledge Type: Core Knowledge, Sensory Abilities, Reading) repeated measures ANOVA revealed no main effect of Wording, $F(3, 198) = 0.87$, $p = .46$, or Wording x Knowledge Type interaction, $F(6, 396) = 1.27$, $p = .27$. Across all wording conditions participants gave primarily learning-based responses for the core abilities and for reading (all *p*'s < .02), but gave almost no learning-based responses for seeing or hearing (all *p*'s < .001).

We also examined the relationship between responses on the core knowledge items and participants' gender, age, level of education, and parental status. A univariate linear regression with Age Onset Estimates as outcome variable and Wording, Gender, Age, Education, and Parental Status as predictors revealed no significant predictors: Wording ($\beta = -0.002$, $p = .99$), Gender ($\beta = 0.08$, $p = .45$), Age ($\beta = -0.13$, $p = .22$), Education ($\beta = -0.05$, $p = .62$), Parental Status ($\beta = 0.12$, $p = .25$). A univariate linear regression with percentage of Learning-based Explanations as outcome variable and Wording, Gender, Age, Education, and Parental Status as predictors revealed that Gender ($\beta = -0.21$, $p = .05$) was a significant predictor; male participants offered fewer learning-based explanations (*M*: 66%, 95% CI [61%, 72%]) than female participants (*M*: 76%, 95% CI [71%, 81%]). Wording ($\beta = 0.16$, $p = .12$), Age ($\beta = -0.18$, $p = .08$), Education ($\beta = 0.09$, $p = .38$), and Parental Status ($\beta = 0.08$, $p = .45$) did not predict participants' Learning-based Explanations.

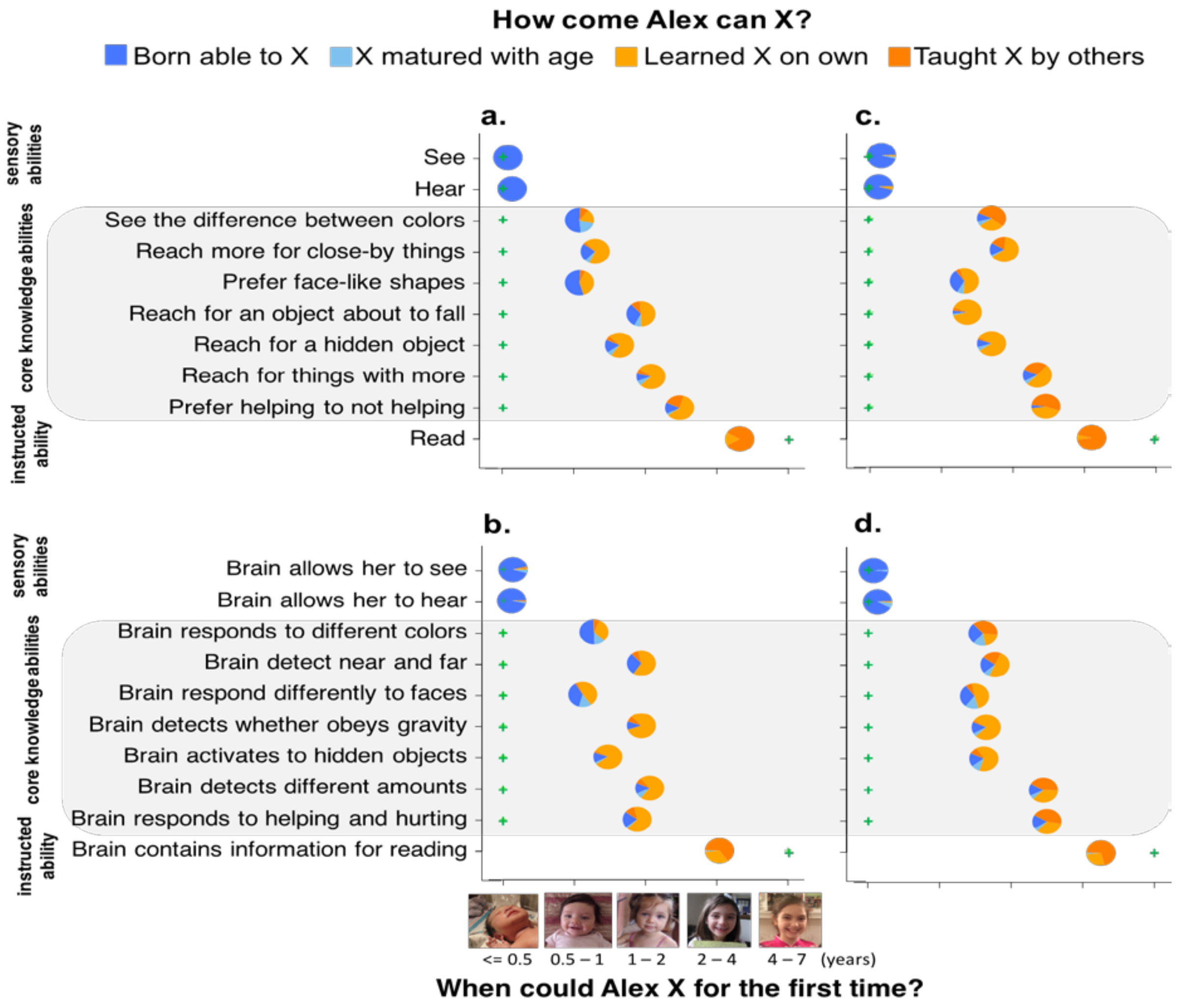


**Fig. S2.** Mean responses in Experiment 1c: a: observable behavior; b: brain activity; c: epistemic state; d: Experiment 1 replication.

## Experiment 2

### Additional Coding Information

Coding and analyses were as in Experiment 1. Inter-coder reliability was 96%.

### Additional Results

In addition to the results reported in the main text, we examined the relationship between participants' responses on the core knowledge items and their gender, age, level of education,

parental status, and religiosity. A univariate linear regression with participants' Age Onset Estimates as outcome variable and Gender, Age, Education, Parental Status, and Religiosity as predictors revealed no significant predictors: Gender ($\beta$ = -0.04, $p$ = .73), Age ($\beta$ = -0.002, $p$ =.98), Education ($\beta$ = -0.08, $p$ =.47), Parental Status ($\beta$ = -0.06, $p$ =.61), Religiosity ($\beta$ = -0.12, $p$ =.26). A univariate linear regression with participants' percentage of Learning-based Explanations as outcome variable and Gender, Age, Education, Parental Status, and Religiosity as predictors revealed Gender ($\beta$ = 0.33, $p$ = .003) as the only significant predictor, with male participants offering more learning-based explanations (*M*: 84%, 95% CI [78%, 90%]) than female participants (*M*: 65%, 95% CI [51%, 80%]). Age ($\beta$ = 0.11, $p$ =.35), Education ($\beta$ = 0.01, $p$ =.92), Parental Status ($\beta$ = 0.04, $p$ =.75), and Religiosity ($\beta$ = 0.03, $p$ =.77) did not predict participants' Learning-based Explanations.

## Experiment 3

### Additional Coding Information

Coding and analyses were as in Experiment 1, except that participants' yes/ no responses to the question of whether a newborn could X were analyzed in place of age of ability onset. Inter-coder reliability was 97%.

### Additional Results

In addition to the results reported in the main text, we examined the relationship between participants' responses on the core knowledge items and their gender, age, level of education, and parental status. Analyses were conducted separately for the Human and Animal conditions.

To examine participants' beliefs about human abilities, we conducted a univariate linear

regression with the percentage of participants' endorsements of a newborn human as having an ability as outcome variable and Gender, Age, Education, and Parental Status as predictors. This revealed Parental Status (β = -0.23, *p* =.03) as the only significant predictor; participants who reported having children were less likely to attribute core abilities to newborn infants (*M*: 29%, 95% CI [20%, 38%] compared to participants who reported having no children (*M*: 43%, 95% CI [36%, 50%]). Gender (β = 0.13, *p* = .22), Age (β = -0.01, *p* = .90) and Education (β = -0.14, *p* =.17) did not predict participants' responses. A univariate linear regression with participants' percentage of Learning-based Explanations as outcome variable and Gender, Age, Education, and Parental Status as predictors revealed that Gender (β = -0.10, *p* = .33), Age (β = 0.09, *p* = .41), Education (β = 0.19, *p* =.08), and Parental Status (β = 0.21, *p* =.06) were not significant predictors.

To examine participants' beliefs about animal abilities, we conducted a univariate linear regression with the percentage of participants' endorsements of a newborn animal as having an ability as outcome variable and Gender, Age, Education, and Parental Status as predictors. This revealed none of the variables as significant predictors: Gender (β = -0.02, *p* = .88), Age (β = -0.09, *p* = .40), Education (β = 0.14, *p* =.17), Parental Status (β = -0.14, *p* =.20). A univariate linear regression with participants' percentage of Learning-based Explanations as outcome variable and Gender, Age, Education, and Parental Status as predictors revealed Education (β = -0.25, *p* =.02) as a significant predictor. Participants who reported having completed at least a college level degree offered fewer learning-based explanations (*M*: 19%, 95% CI [10%, 28%]) than participants with no college education (*M*: 33%, 95% CI [28%, 39%]). Gender (β = -0.007, *p* = .94). Age (β = 0.10, *p* = .34), and Parental Status (β = 0.11, *p* =.32) were not significant predictors.

**Experiment 4**

Additional Coding Information

An experienced observer coded all transcribed responses using the criteria from Experiment 1. A second observer coded 50% of all data; inter-coder reliability was 98%.

Additional Results

In addition to the results reported in the main text, we examined the relationship between children's responses on the core knowledge items and their gender and age.

A univariate linear regression with participants' Age Onset Estimates as outcome variable and Gender and Age as predictors revealed Age as the only significant predictor ($\beta$ = -0.30, *p* = .006). Children's onset estimates of core abilities decreased with age. Gender ($\beta$ = 0.021, *p* = .85) did not significantly predict participants' responses. A univariate linear regression with participants' percentage of Learning-based Explanations as outcome variable and Gender and Age as predictors revealed neither as a significant predictor: Gender ($\beta$ = 0.048, *p* = .67); Age ($\beta$ = -0.12, *p* =.28).

**Experiment 5**

Additional Coding Information

Coding and analyses were as in Experiment 1. Inter-coder reliability was 95%.

Additional Results

In addition to the results reported in the main text, we examined the effects of gender, age, education level, parental status, and participants' field of study.

A univariate linear regression with participants' Age Onset Estimates as outcome variable and Field of Study, Gender, Age, Education, and Parental Status as predictors revealed that Field of Study was a significant predictor (β = 0.12, $p = .02$), with mind scientists offering earlier age onset estimates (*M*: 1.04 years, 95% CI [0.98, 1.10]) than other academics (*M*: 1.10 years, 95% CI [1.04, 1.17]). Age was also a significant predictor (β = -0.17, $p = .01$). Age onset estimates decreased with participants' age. Gender (β = -0.07, $p = .18$), Education (β = 0.01, $p$ =.80), and Parental Status (β = -0.02, $p$ =.71) did not significantly predict participants' responses. A univariate linear regression with participants' percentage of Learning-based Explanations as outcome variable and Field of Study, Gender, Age, Education, and Parental Status as predictors revealed Field of Study as a significant predictor (β = 0.23, $p < .001$); with mind scientists offering fewer learning-based explanations (*M*: 59%, 95% CI [55%, 63%]) than other academics (*M*: 70%, 95% CI [67%, 73%]). Gender was also a significant predictor (β = -0.16, $p = .002$); male participants offered fewer learning-based explanations (*M*: 59%, 95% CI [54%, 64%]) than female participants (*M*: 67%, 95% CI [64%, 70%]). Age (β = -0.11, $p$ =.11), Education (β = -0.03, $p$ =.52), and Parental Status (β = 0.10, $p$ =.12) were not significant predictors.

**Table S1.** Survey questions in Experiments 1, 2, 4, and 5

| Ability | Age Onset Question | Ability Origin Question |
|---|---|---|
| **See** | Alex can see things with her eyes. When could Alex see with her eyes for the first time? | How come she can see? |
| **Hear** | When there is a sound close by, Alex can hear it. When could Alex hear sounds for the first time? | How come she can hear? |
| **Discriminate colors** | When seeing a red flower and a blue flower, Alex can tell that they are different colors. Alex can tell colors apart. When could Alex tell colors apart for the first time? | How come she can tell colors apart? |
| **Discriminate distances** | When there is a car approaching, Alex can tell that the car is getting closer. Alex can tell what is near and what is far. When could Alex tell near and far for the first time? | How come she can tell near and far? |
| **Prefer faces to non-faces** | If Alex sees the above pictures, Alex thinks that the picture on the left looks a bit more like a face. Alex can tell whether something looks like a face or not. When was the first time Alex could tell whether something looks like a face? | How come she can tell whether something looks like a face? |
| **Think unsupported objects will fall** | When Alex sees someone hold an object and then drop it, Alex thinks the object will fall. Alex thinks objects will fall if we let go of them. When could Alex think that for the first time? | How come she can think that objects will fall if we let go of them? |
| **Think hidden objects are still there** | If Alex sees a toy being hidden in a box, she will think the object is still there even though she can no longer see it. When could Alex think that for the first time? | How come she can think that hidden objects will still be there? |
| **Discriminate quantities** | If Alex sees two cookies, one with 5 chocolate chips in it and one with 20 chocolate chips in it, she can tell which cookie has more chocolate chips without counting. Alex can tell which has more. When could Alex tell which has more for the first time? | How come she can tell which has more? |
| **Prefer helping to not helping** | If Alex sees a turtle that is upside down and struggling to get on its feet, she thinks that she should help the turtle. Alex thinks that helping is the right thing to do. When could Alex think that for the first time?[1] | How come she can think that helping is right?[1] |
| **Read** | Alex can read books. When could Alex read for the first time? | How come she can read? |

[1] In Experiment 1, the wording of the social evaluation item (e.g., "How come she can think that helping is right?") used a rich interpretation of the results reported by (*7*). To ensure that this wording did not determine the observed effects, we replicated Experiment 1 with a separate group of participants (N = 101, *M*: 32.97 years old; 61 female). These participants completed the survey as in Experiment 1, but with the social evaluation item changed to "Alex prefers someone who helps the turtle over someone who hurts the turtle. When could Alex first prefer someone who helps?" and "How come she can prefer someone who helps?" As in Experiment 1, participants overestimated the age of onset of social evaluation (*M*: 2.56 years; 95% CI [2.29, 2.84]), and gave predominantly learning-based explanations (*M*: 83.10%, binomial exact test $p < .001$). This modified wording was used for the social evaluation item in Experiments 1c and 2.

**Table S2.** Age of participants demonstrating target abilities in published findings.

| Ability | Participants' average age | Citation |
|---|---|---|
| **See** | 2.7 weeks | Brown, A. M., & Yamamoto, M. (1986). Visual Acuity in Newborn and Preterm Infants Measured With Grating Acuity Cards. *American Journal of Ophthalmology*, *102*(2), 245–253. https://doi.org/10.1016/0002-9394(86)90153-4 |
| **Hear** | 0 days | Northern, J. L., & Downs, M. P. (2002). *Hearing in Children*. Lippincott Williams & Wilkins. |
| **Discriminate colors** | 4 months | Bornstein, M. H., Kessen, W., & Weiskopf, S. (1976). Color vision and hue categorization in young human infants. *Journal of Experimental Psychology: Human Perception and Performance*, *2*(1), 115–129. https://doi.org/10.1037/0096-1523.2.1.115 |
| **Discriminate distances** | 2 days | Slater, A., Mattock, A., & Brown, E. (1990). Size constancy at birth: Newborn infants' responses to retinal and real size. *Journal of Experimental Child Psychology*, *49*(2), 314–322. https://doi.org/10.1016/0022-0965(90)90061-C |
| **Prefer faces to non-faces** | 168 hours | Farroni, T., Johnson, M. H., Menon, E., Zulian, L., Faraguna, D., & Csibra, G. (2005). Newborns' preference for face-relevant stimuli: Effects of contrast polarity. *Proceedings of the National Academy of Sciences*, *102*(47), 17245–17250. https://doi.org/10.1073/pnas.0502205102 |
| **Think unsupported objects will fall** | 3 months | Baillargeon, R. (1995). Physical Reasoning in Infancy. In M. S. Gazzaniga (Ed.), *The Cognitive Neurosciences* (pp. 181–204). MIT Press. |
| **Think hidden objects are still there** | 3.5 months | Baillargeon, R. (1987). Object Permanence in 3 1/2- and 4 1/2-Month-Old Infants. *Developmental Psychology*, *23*(5), 655–664. |
| **Discriminate quantities** | 49 hours | Izard, V., Sann, C., Spelke, E. S., & Streri, A. (2009). Newborn infants perceive abstract numbers. *Proceedings of the National Academy of Sciences*, *106*(25), 10382–10385. https://doi.org/10.1073/pnas.0812142106 |
| **Prefer helping to not helping** | 6 months | Hamlin, J. K., Wynn, K., & Bloom, P. (2007). Social evaluation by preverbal infants. *Nature*, *450*(7169), 557–559. https://doi.org/10.1038/nature06288 |
| **Read** | 7 years | Hasbrouck Jan, & Tindal Gerald A. (2011). Oral Reading Fluency Norms: A Valuable Assessment Tool for Reading Teachers. *The Reading Teacher*, *59*(7), 636–644. https://doi.org/10.1598/RT.59.7.3 |

**Table S3.** Survey questions in Experiment 1c

| | A. Observable Behavior | | B. Brain Activity | | C. Epistemic State | |
|---|---|---|---|---|---|---|
| **Ability** | **Age Onset Question** | **Ability Origin Question** | **Age Onset Question** | **Ability Origin Question** | **Age Onset Question** | **Ability Origin Question** |
| **See** | Alex can see things with her eyes. When could Alex see with her eyes for the first time? | How did Alex become able to see? | Alex can see things with her eyes. When did Alex's brain first allow her to see anything? | How did her brain come to allow her to see? | Alex knows how to see things with her eyes. When could Alex know how to see for the first time? | How did Alex come to know how to see? |
| **Hear** | When there is a sound close by, Alex can hear it. When could Alex hear sounds for the first time? | How did Alex become able to hear? | When there is a sound close by, Alex can hear it. When did Alex's brain first allow her to hear anything? | How did her brain come to allow her to hear? | When there is a sound close by, Alex can hear it. When could Alex know how to hear for the first time? | How did Alex come to know how to hear? |
| **Discriminate colors** | When seeing a red flower and a blue flower, Alex can see that they have different colors. Alex can see the difference between colors. When could Alex see the difference between colors for the first time? | How did Alex come to see the difference between colors? | Alex's brain responds differently to the red flower than the blue flower. Her brain responds differently to different colors. When was the first time her brain responded differently to different colors? | How did her brain come to respond to different colors? | When seeing a red flower and a blue flower, Alex knows that they have different colors. Alex knows how to tell different colors apart. When could Alex know how to tell colors apart for the first time? | How did Alex come to know how to tell colors apart? |
| **Discriminate distances** | When there is an object approaching, Alex can reach for the object when the object gets closer. Alex reaches for close-by things rather than far-away things. When could Alex first reach more for close-by things than far-away things? | How did Alex come to reach more for close-by things than far-away things? | When there is a car approaching, Alex's brain detects that the car is getting closer. Alex's brain can detect the difference between nearby things and faraway things. When was the first time her brain could tell the difference between nearby things and faraway things? | How did her brain come to detect near and far? | When there is a car approaching, Alex knows that the car is getting closer. Alex knows the difference between nearby things and faraway things. When could Alex first know the difference between nearby things and faraway things for the first time? | How did Alex come to know the difference between nearby things and faraway things? |

**Table S3.** Survey questions in Experiment 1c (continued)

| | **A. Observable Behavior** (continued) | | **B. Brain Activity** (continued) | | **C. Epistemic State** (continued) | |
|---|---|---|---|---|---|---|
| **Ability** | **Age Onset Question** | **Ability Origin Question** | **Age Onset Question** | **Ability Origin Question** | **Age Onset Question** | **Ability Origin Question** |
| **Prefer faces to non-faces** | If Alex sees the above pictures, Alex looks longer at the picture on the left. Alex prefers to look at face-like shapes than at other shapes. When was the first time Alex preferred to look at face-like shapes over other shapes? | How did Alex come to prefer face-like shapes over other shapes? | If Alex sees the above pictures, her brain activates differently for the picture on the left than for the picture on the right. Her brain responds differently to things that look sort of like faces versus other objects. When was the first time her brain responded differently to face-like things? | How did her brain come to respond differently to face-like things versus other objects? | If Alex sees the above pictures, Alex knows that the picture on the left looks a bit more like a face. Alex knows whether something looks sort of like a face or not. When could Alex know that for the first time? | How did Alex come to know whether something looks like a face or not? |
| **Think unsupported objects will fall** | When Alex sees someone hold an object and then drop it, Alex reaches out for the object because it is about to fall. Alex can reach for things that are about to fall. When could Alex first reach out for an object that is about to fall? | How did Alex come to reach out for an object that's about to fall? | When Alex sees someone about to drop an object, her brain activates if the object appears to hover in mid-air, instead of falling down. Her brain detects it when something disobeys gravity. When was the first time her brain detected if something obeys gravity? | How did her brain come to detect whether something obeys gravity? | When Alex sees someone hold an object and then drop it, Alex knows that the object will fall. Alex knows that objects will fall if we let go of them. When could Alex know that for the first time? | How did Alex come to know that objects will fall if we let go of them? |
| **Think hidden objects are still there** | If Alex sees a toy being hidden in a box, she reaches for the toy even though she can no longer see it. When could she first reach for a toy that she could no longer see? | How did Alex come to reach for a hidden object? | After Alex sees a toy being hidden in a box, her brain still activates even though she can no longer see the toy. Her brain activates even to hidden objects. When was the first time her brain activated in response to a hidden object? | How did her brain come to activate to hidden objects? | If Alex sees a toy being hidden in a box, she knows that the toy is still there even though she can no longer see it. Alex knows that hidden objects can still be there even though we cannot see them. When could Alex first know that hidden objects can still be there? | How did Alex come to know that hidden things can still be there? |

**Table S3.** Survey questions in Experiment 1c (continued)

| | **A. Observable Behavior** (continued) | | **B. Brain Activity** (continued) | | **C. Epistemic State** (continued) | |
|---|---|---|---|---|---|---|
| **Ability** | **Age Onset Question** | **Ability Origin Question** | **Age Onset Question** | **Ability Origin Question** | **Age Onset Question** | **Ability Origin Question** |
| **Discriminate quantities** | If Alex sees two cookies, one with 5 chocolate chips in it and one with 20 chocolate chips in it, she reaches for the cookie with more chocolate chips, without counting. Alex reaches for the amount that's more. When could she reach for something with more for the first time? | How did Alex come to be able to reach for things with more? | If Alex sees two cookies, one with 5 chocolate chips in it and one with 20 chocolate chips in it, her brain responds differently to the two amounts, without counting. When was the first time her brain detected different amounts? | How did her brain come to detect different amounts? | If Alex sees two cookies, one with 5 chocolate chips in it and one with 20 chocolate chips in it, she knows which cookie has more chocolate chips without counting. Alex knows which is more. When could Alex know that for the first time? | How did Alex come to know which has more? |
| **Prefer helping to not helping** | Alex sees a turtle that is upside down and struggling to get on its feet. If she then sees one person help the turtle get back on its feet, and another person let the turtle continue to struggle, Alex prefers the person who helped over the person who did not help. When was the first time Alex preferred helping? | How did Alex come to prefer helping? | Alex sees a turtle that is upside down and struggling to get on its feet. If she then sees someone help the turtle get back on its feet, her brain responds differently than it would if she sees someone hurting the turtle. Her brain responds differently to helping and hurting. When was the first time her brain responded differently to helping and hurting? | How did her brain come to respond to helping and hurting? | Alex sees a turtle that is upside down and struggling to get on its feet. If she then sees one person help the turtle get back on its feet, and another person let the turtle continue to struggle, Alex knows that helping was the right thing to do. When could Alex know that for the first time? | How did Alex come to know that helping is the right thing to do? |
| **Read** | Alex can read books. When could Alex read for the first time? | How did Alex become able to read? | Alex can read books. When was the first time Alex's brain contained all of the information she needed to read a book? | How did her brain come to contain the information needed for reading a book? | Alex can read books. When did Alex know how to read for the first time? | How did Alex come to know how to read? |
